# A Phase III Prospective Active and Placebo-Controlled Randomized Trial of Vilazodone in the Treatment of Major Depressive Disorder

**DOI:** 10.7759/cureus.16689

**Published:** 2021-07-28

**Authors:** Shubhadeep Sinha, Sreenivasa Chary, Pankaj Thakur, Leela Talluri, Mohan Reddy, Kamal K Verma, Pradeep Saha, Vijaya B Gupta, Kaja A Ramaiah, Siquafa Z Khanum

**Affiliations:** 1 Clinical Development and Medical Affairs, Hetero Labs Limited, Hyderabad, IND; 2 Psychiatry, Sardar Patel Medical College, Bikaner, IND; 3 Psychiatry, Institute of Post-Graduate Medical Education and Research, Kolkata, IND; 4 Psychiatry, Rajiv Gandhi Institute of Medical Sciences, Srikakulam, IND; 5 Psychiatry, Sri Manasa Psychiatrist Hospital, Vijayawada, IND; 6 Psychiatry, Nirmal Hospital, Jhansi, IND

**Keywords:** antidepressant, major depressive disorder, selective serotonin reuptake inhibitor, vilazodone, 5-ht1a partial agonist

## Abstract

Background

Depression is a leading cause of psychiatric morbidity in the modern world, and the introduction of selective serotonin reuptake inhibitors (SSRIs) is a revolution in the treatment of depression. Vilazodone, a novel SSRI and 5-HT1A partial agonist, received FDA approval in 2011 to treat the major depressive disorder (MDD) in adults. This study conducted in India aimed to evaluate the efficacy and safety of vilazodone when compared to escitalopram or placebo in patients with MDD.

Methods

This was a prospective, multicentre, randomized, comparative study of 375 participants over eight weeks of treatment with either vilazodone (10-40mg/day) or escitalopram (10-40 mg/day) or placebo in adult patients with MDD. Primary efficacy was assessed using the Hamilton Rating Scale for Depression (HAM-D-17); secondary efficacy was assessed using the Montgomery-Asberg Depression Rating Scale (MADRS) score and Hamilton Anxiety Scale (HAM-A) score. Safety parameters included adverse events (AEs), clinical laboratory results, vital signs, electrocardiogram ( ECG), and Columbia-Suicide Severity Rating Scale (C-SSRS).

Results

Mean change in the HAM-D-17 total score from baseline to week 8 for vilazodone, escitalopram, and placebo-treated patients in intent-to-treat (ITT) population was: -18.9 (± 7.49), -17.8 (± 6.06), and -7.4 (± 6.32); in ITT population (with Last Observation Carried Forward( LOCF) imputation) was: -17.9 (± 7.71), -17.4 (± 6.19), and -6.4 (± 6.84), and in per-protocol (PP) population was: -19.1 (± 7.20), -17.8 (± 6.08), and -7.7 (± 6.29), respectively. The upper limit of 95% CI (0.56 (ITT); 0.90 (ITT with LOCF Imputation); 0.23 (PP)) of difference in HAM-D-17 between vilazodone 40mg and escitalopram 40mg, which is lower than the defined non-inferiority margin (3.56), proving non-inferiority. The difference between vilazodone 40mg, escitalopram 40mg, and the placebo was statistically significant (p<0.0001). No deaths or serious adverse events were reported in this study.

Conclusion

Vilazodone demonstrated comparable efficacy to escitalopram and superior efficacy over the placebo in the treatment of MDD.

## Introduction

Major depressive disorder (MDD) is a prevalent psychiatric ailment worldwide, affecting more than 350 million people of all ages [1]. Over 30% of patients with MDD do not achieve an adequate response and remission [2] after treatment. Non-adherence and premature treatment discontinuation are fairly com­mon [3] and can be traced back to limited efficacy or delayed onset as well as intoler­able adverse events, especially weight gain and sexual dysfunction [4]; SSRIs are the most commonly prescribed first-line antidepressants [5] for the treatment of MDD. Vilazodone is the first member of the serotonin partial agonist-reuptake inhibitor (SPARI) class of medications, which combines serotonin reuptake inhibition with 5-HT1A partial agonist action, approved by the Food and Drug Administration (FDA) for the treatment of MDD in adults [6]. It has potential benefits, including a faster onset of action, greater efficacy, and better tolerability. Vilazodone combines serotonin reuptake inhibition and buspirone-like anxiolytic mechanism, which could be an effective and well-tolerated class of drugs for patients with MDD symptoms and anxiety disorders. It might have fewer sexual side effects than conventional SSRIs [7]. In a 10-week placebo-controlled and active-controlled clinical trial (NCT01473381) conducted in 54 psychiatry centres in the USA, adult patients with MDD were randomized to 1:1:1:1 (vilazodone 20 or 40mg/day, citalopram 40mg/day, or placebo). Montgomery-Asberg Depression Rating Scale (MADRS) and Clinical Global Impression-severity (CGIS) score change from baseline to week 10 were significantly greater for vilazodone 20mg/day, vilazodone 40mg/day, and citalopram than for placebo. The most common adverse events were diarrhoea, nausea, vomiting, and insomnia. Improved sexual function was observed in all the groups. Vilazodone 20 and 40 mg/day demonstrated efficacy and tolerability in the treatment of MDD [8].

Among the SSRIs, escitalopram potentiates its binding, raising the possibility of increasing effects with increasing doses. It is currently prescribed for the treatment of major depression at doses of 5-40 mg. In a study conducted by Wade et al. (2011), patients were treated with escalating doses of escitalopram up to 50mg for 32 weeks until remission of MADRS ≤ 8 or failure to tolerate the dose. The percentage of patients who required a 50mg dose to achieve remission was 38%. The median time to remission was 24 weeks, and the median dose in remission was 30mg [9]. Qi et al. (2017) demonstrated the efficacy, tolerability, and adherence to high-dose escitalopram at 40mg/day in a 12 weeks’ study [10]. In another randomized trial by Zuilhof et al. (2018), escitalopram was significantly superior (on average 30mg/day) over bupropion (450mg/day) at the end of 12 weeks of initial treatment (remission rates of 52% vs 34%, respectively). Escitalopram can be administered up to 40mg/day and bupropion up to 450mg/day [11].

The clinical efficacy and safety data of vilazodone in Indian patients with MDD were not generated earlier. There is also a significant need for effective medications for patients with MDD in India. Therefore, this clinical study was designed to evaluate the efficacy, safety, and tolerability of vilazodone in treating adult Indian patients with MDD. Escitalopram was included as active control for assay sensitivity.

## Materials and methods

Study design

This 8-week, multicenter, randomized, open-label, active-controlled, and placebo-controlled study was conducted at 11 Multispecialty Hospitals’ Psychiatry Departments across India between December 2013 and May 2015 in compliance with the Declaration of Helsinki: Ethical Principles for Medical Research Involving Human Patients; statutory provisions prescribed under Schedule ‘Y’ to the Drugs & Cosmetic Rules, 1945 as amended; Ethical Guidelines for Biomedical Research, 2006 by Indian Council of Medical Research, and principles for Good Clinical Practice (GCP) as well as the Indian GCP guidelines. The trial was approved by the Drugs Controller General of India, Central Drug Standard Control Organization, India, and registered with the Clinical Trial Registry India (CTRI/2013/11/004127). The study commenced with approval from the institutional ethics committee at each study centre, and all patients provided written informed consent. All efficacy assessments were conducted in person at each investigative centre by experienced and qualified psychiatrists (investigators and co-investigators) at baseline and subsequent visits. The study comprised screening, up to a 4-week washout period (depending on the half-life of ongoing drugs), baseline visit on day 1, and an initial titrated-dose period of 2 weeks followed by a fixed-dose treatment period of six weeks.

Participants

In this study, 375 patients with mild-to-moderate severity of depression (as per DSM-IV) were randomly assigned by computer-generated numbers to vilazodone (10-40 mg/day; n = 126), escitalopram (10-40 mg/day; n = 125), or placebo (n = 124). The intent-to-treat (ITT) population consisted of 348 (92.80%) patients: 117 (92.86%) in the vilazodone group, 117 (93.60%) in the escitalopram group, and 114 (91.94%) in the placebo group. The per protocol (PP) population consisted of 307 (81.87%) patients: 103 (81.75%) in the vilazodone group, 111 (88.80%) in the escitalopram group, and 93 (75.00%) in the placebo group.

Inclusion criteria

This study included male and female outpatients (18-65 years of age, inclusive) who met the Diagnostic and Statistical Manual of Mental Disorders, Fourth Edition, Text Revision (DSM-IV-TR) 10 criteria for MDD [12], had an ongoing major depressive episode lasting four or more weeks and up to 24 months, and had a HAM-D-17 [13] total score of up to 20. Selected patients must have general ocular health. Female patients of childbearing potential were required to have a negative β-hCG pregnancy test and, using a reliable method of contraception, were allowed to participate in the study.

Exclusion criteria

Patients with a decrease in HAM-D-17 score by less than or equal to 25% between screening and baseline, patients who were actively suicidal or likely to require hospitalization during the trial, those who had undergone electroconvulsive therapy within the last six months of screening, those who were likely to require other psychotropic medications and central nervous system active drugs or patients with medical conditions likely to interfere with study conduct or to confound with the results, or to endanger patient well-being were excluded. Patients who had participated in an investigational drug trial within the past 30 days were excluded.

Exclusion criteria also included the history of nonresponse or known hypersensitivity to SSRIs and patients with nonresponse to adequate treatment with two or more consecutive antidepressants. Patients taking migraine medications with a serotonergic mechanism of action or NSAIDs or drugs affect coagulation or CYP3A4 inhibitors/ inducers or monoamine oxidase (MAO) inhibitors (or within 14 days of stopping MAO inhibitors) or patients previously treated with vilazodone were also excluded. Substance abuse as per DSM-ITR criteria within three months before the screening visit or substance dependence within six months before the screening visit was also exclusionary.

Efficacy and safety assessments

The primary efficacy endpoint was the change from baseline to week 8 in the HAM-D-17 total score. Secondary efficacy endpoints were changes in the Montgomery-Asberg Depression Rating Scale ( MADRS) and Hamilton Anxiety Scale (HAM-A) total score from baseline to week 8. Safety was assessed by adverse event reports, physical examination, laboratory values, vital sign measurements, ECGs, ophthalmic evaluations, and Columbia-Suicide Severity Rating Scale (C-SSRS) score.

Statistical analyses

A sample size of at least 104 evaluable patients per group was sufficient to prove the non-inferiority (NI Margin = - 20% of the control value) of vilazodone compared with escitalopram with 80% power and at a 5% level of significance. Assuming the dropout rate of 20%, 125 patients were planned in each group to achieve 312 evaluable patients. The central randomization scheme was generated based on permuted blocks of size four and a ratio of 1:1:1 in the treatment groups using the statistical analysis software, SAS® (SAS Institute Inc., NC, USA). Analysis was performed on ITT, ITT with LOCF, and PP populations. Primary endpoint data were analyzed using the Wilcoxon signed-rank test, Mann-Whitney test, and ANCOVA method, as appropriate. Categorical data are presented as the absolute number/percentage of patients.

In contrast, quantitative data are presented as mean ± standard deviation (SD) or median (range), 95% CI confidence interval, and mean change from baseline to week 8. Statistical significance was defined as a two-sided p-value < 0.05. Descriptive statistics were used for all the safety parameters. Statistical analysis was performed using SAS software (version 9.4; SAS Institute Inc., Cary, NC, USA). Adverse events were coded using Version 15.0 of the Medical Dictionary for Regulatory Activities (MedDRA).

## Results

Patient disposition and characteristics

**Figure 1:**
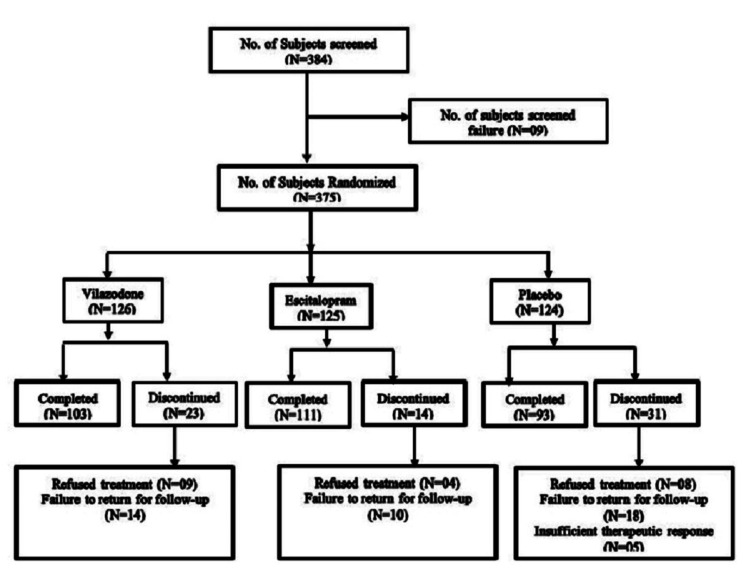
Disposition of Patients N is the number of patients in the specified treatment

Overall, 375 patients were randomly assigned in a ratio of 1:1:1 to vilazodone, escitalopram, and placebo (Fig 1). Of the 375 patients randomized, 307 (82.13%) patients (vilazodone group (103: 81.75%), escitalopram group (111: 88.80%), and placebo group (93: 75.00%)) completed the study (Fig 1).

The ITT population comprised 348 patients, and the PP population comprised 307 patients. Patient disposition and demographics are presented in table 1.

**Table 1:** Demographics and other baseline characteristics BMI calculated as weight (kg)/height ² (m²); N is the number of patients in a specified treatment; n is the number of patients in a specified category

Demography Parameters	Vilazodone (N=126) n (%)	Escitalopram (N=125) n (%)	Placebo (N=124) n (%)	Overall (N=375) n (%)
Age (Years) Mean (SD)	36 (11)	35 (10)	37 (11)	36 (11)
Male (%)	65 (51.59%)	75 (60.00%)	76 (61.29%)	216 (57.60%)
Female (%)	61 (48.41%)	50 (40.00%)	48 (38.71%)	159 (42.40%)
Height (cm) Mean (SD)	162.01 (9.86)	162.00 (8.96)	161.82 (9.32)	161.94 (9.36)
Body Weight (Kg) Mean (SD)	60.62 (9.62)	60.72 (11.20)	60.23 (10.84)	60.52 (10.54)
BMI (kg/m^2^) Mean (SD)	23.13 (3.28)	23.13 (3.75)	23.07 (4.04)	23.11 (3.69)

Analysis of Efficacy

Primary Efficacy Outcome

Mean change in Hamilton-D -17 total score from baseline to week 8 in the vilazodone, escitalopram, and placebo groups in ITT population was -18.9 (± 7.49), -17.8 (± 6.06), and -7.4 (± 6.32); ITT population (with LOCF imputation) was -17.9 (± 7.71), -17.4 (± 6.19), and -6.4 (± 6.84), and PP population was -19.1 (± 7.20), -17.8 (± 6.08), and -7.7 (± 6.29), respectively. The upper bound of the one-sided 95% CI of the difference in change from baseline in the HAM-D-17 score between vilazodone, escitalopram, and the placebo in the ITT population was 0.56; ITT with LOCF imputation population was 0.90, and PP population was 0.23, which was lower than the non-inferiority margin of 3.56 (20% of the control value), proving the non-inferiority of vilazodone to escitalopram. The difference in change from baseline in the HAM-D-17 score between the vilazodone and placebo groups was statistically significant (P < 0.0001), favouring vilazodone over the placebo (Table 2).

**Table 2:** Mean change from baseline to week 8 in the Hamilton Rating Scale for Depression (HAM-D-17) total score N = number of patients in the treatment group; n = number of patients with non-missing values at baseline and post-baseline. Note: ITT = Intend-To-Treat; PP = per protocol. p-values calculated using the ANCOVA model: Change from baseline to baseline + treatment + site number.  **p- value highly significant (˂ 0.0001)

Treatment	n	Mean ± SD	Vilazodone Vs Escitalopram 95% Confidence Interval	Vilazodone Vs Placebo (p-value)	Escitalopram Vs Placebo (p-value)
ITT population					
Vilazodone (N=117)	104	-18.9 ± 7.49	(-1.73, 0.56)	<0.0001	<0.0001
Escitalopram (N=117)	112	-17.8 ± 6.06			
Placebo (N=114)	97	-7.4 ± 6.32			
ITT population (with LOCF Imputation)					
Vilazodone (N=117)	117	-17.9 ± 7.71	(-1.43, 0.90)	<0.0001	<0.0001
Escitalopram (N=117)	117	-17.4 ± 6.19			
Placebo (N=114)	114	-6.4 ± 6.84			
PP Population					
Vilazodone (N=103)	103	-19.1 ± 7.20	(-1.91, 0.23)	<0.0001	<0.0001
Escitalopram (N=111)	111	-17.8 ± 6.08			
Placebo (N=93)	93	-7.7 ± 6.29			

Secondary Efficacy Outcomes

**Figure 2:**
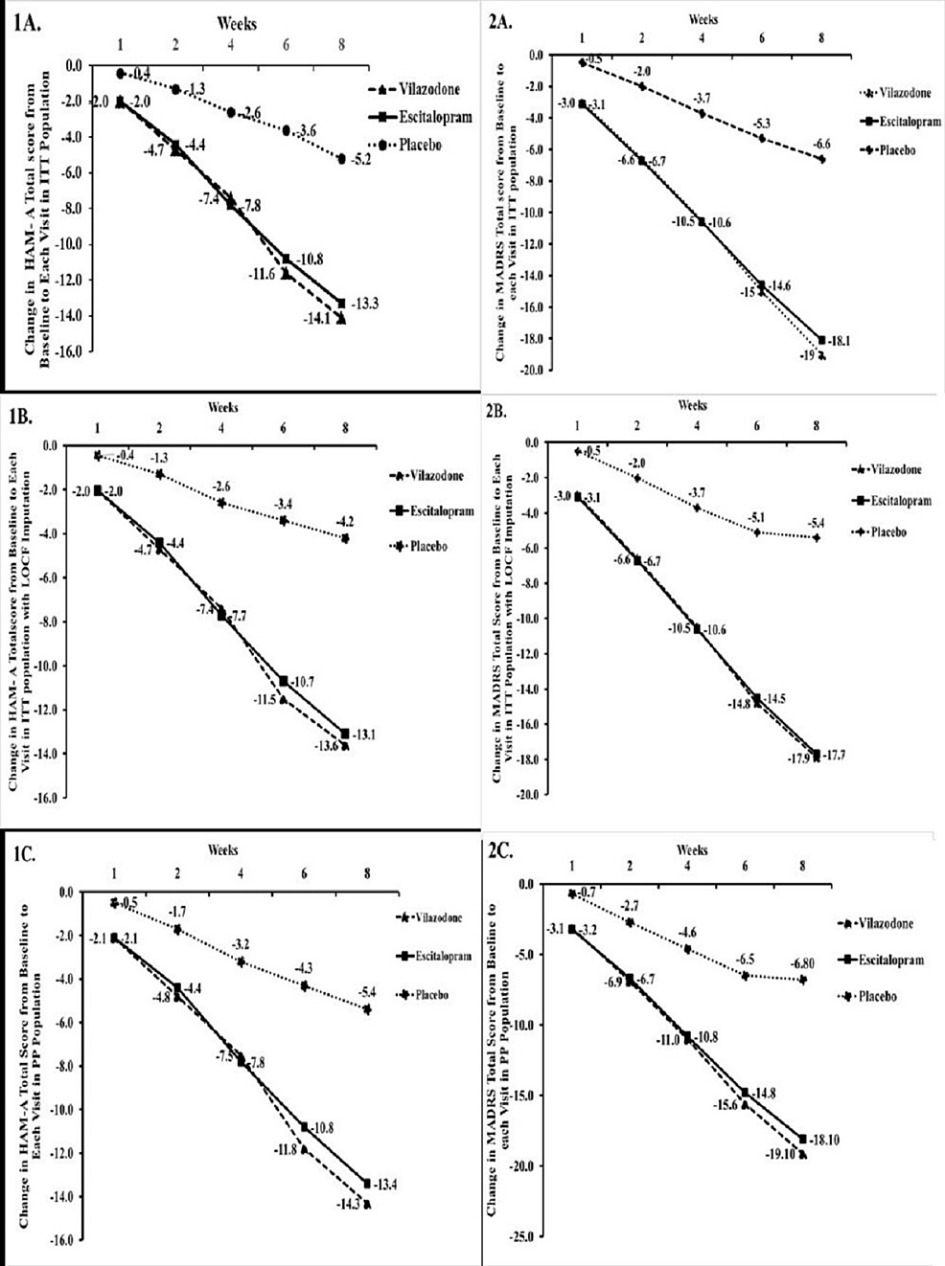
Change in HAM-A total score & MADRS total score from baseline to each visit in ITT, ITT with LOCF imputation, and PP population HAM-A- Hamilton Anxiety Scale, MADRS- Montgomery-Asberg Depression Rating Scale, ITT- intent-to-treat, ITT with LOCF- Intention-to-Treat with Last Observation Carried Forward, PP- Per-protocol

The mean change in MADRS score from baseline to week 8 in the vilazodone, escitalopram, and placebo groups in the ITT population was -19.0 ± 9.42, -18.1 ± 7.51, and -6.6 ± 6.64; ITT population with LOCF imputation was -17.9 ± 9.51, -17.7 ± 7.61, and -5.4 ± 7.10; and PP population was -19.1 ± 9.26, -18.1 ± 7.54, and -6.8 ± 6.63, respectively. The decrease in mean MADRS scores from baseline to week 8 was not statistically significant (p = 0.1677, 0.4799, and 0.0679 in ITT, ITT population with LOCF imputation, and PP population, respectively) between the vilazodone and escitalopram-treated patients, which concluded that vilazodone was therapeutically non-inferior to escitalopram (Figure 2). Vilazodone indicated a significant reduction in the MADRS score compared to the placebo (p < 0.0001), favouring the superiority of vilazodone over the placebo. Mean change in HAM-A total score from baseline to week 8 in the vilazodone, escitalopram, and placebo-treated patients in the ITT population was -14.1 (± 5.30), -13.3 (± 4.89), and -5.2 (±6.42); ITT population (with LOCF Imputation) was 13.62 (± 5.30), -13.15 (± 4.86), and -4.28 (±6.80); PP population was -14.3 (± 5.01), -13.4 (± 4.88), and -5.4 (±6.44), respectively. The decrease in mean HAM-A total score from baseline to week 8 was not statistically significant between vilazodone-treated patients and escitalopram-treated patients (p = 0.0902, 0.2872, 0.0213 in ITT and ITT populations with LOCF imputation, and PP, respectively), which suggests that vilazodone is therapeutically non-inferior to escitalopram (Figure 2). The decrease in the HAM-A score from baseline to week 8 was statistically significant (p < 0.0001) in the vilazodone group compared to the placebo group, favouring the superiority of vilazodone over the placebo.

Safety results

The adverse events (AEs) reported were 57, 54, and 40 in the vilazodone, escitalopram, and placebo treatment groups. In the vilazodone group, with 10, 20, and 40mg of the daily dose, the number of adverse events reported was 16, 17, and 24. In the escitalopram group, with 10, 20, and 40mg of the daily dose, the number of adverse events reported was 20, 8, and 26. In the vilazodone group, the number of AEs related to the investigational product was 1, 4, and 1 with 10, 20 and 40mg vilazodone. Similarly, in the escitalopram group, the number of AEs related to the investigational product was 1, 0, and 5 with 10, 20 and 40mg escitalopram. The most commonly reported adverse events were decreased appetite and dizziness in both the vilazodone and escitalopram groups. All adverse events were mild to moderate. None of the patients discontinued treatment because of adverse events. No deaths or serious adverse events were recorded. No clinically significant ECG findings, vital signs, laboratory findings, physical findings, or other observations related to the study drug's safety were recorded in this study.

## Discussion

This study demonstrated the potential antidepressant activity of vilazodone in patients with MDD. The change in mean HAM-D score from baseline to 8 weeks of study was significantly lowered in both the vilazodone and escitalopram (P < 0.0001) groups. The upper bound of the 95% CI of the mean HAM-D score from baseline to 8 weeks between vilazodone and escitalopram was 0.56 (0.23), a lower non-inferiority margin of 3.56; this proves that vilazodone is non-inferior to escitalopram. Vilazodone-treated patients indicated statistically significant (p < 0.0001) improvement from baseline to the end of treatment in mean MADRS scores compared to placebo. No statistically significant difference in the mean MADRS scores was observed between the active treatment groups. Vilazodone-treated patients indicated statistically significant (p<0.0001) improvement in mean HAM-A scores from baseline to end of treatment compared to placebo. Both vilazodone and escitalopram indicated significant advantages over the placebo in all efficacy measures and indicated a good safety profile with no serious adverse events.

The results obtained in this study are comparable with those reported in previous studies published in the peer-reviewed literature. Khan et al.2011, [14] reported in a phase III trial that during eight weeks of treatment with vilazodone, patients had significantly greater improvements (p = 0.009) according to the MADRS than in the placebo group (intent-to-treat; least squares mean changes: -13.3, -10.8). MADRS response rates were significantly higher with vilazodone than with placebo (44% vs 30%, p = 0.002). In a similar study conducted by Rickels et al.2009, the mean changes in MADRS and HAM-D-17 total scores from baseline to week eight were significantly (p = 0.001 and p = 0.022, respectively) greater with vilazodone than with placebo (Rickels et al.). Significant (p < 0.05) improvements in MADRS and HAM-D-17 scores were noted in week 1, the earliest time point measured. Response rates were significantly higher with vilazodone than with placebo on the MADRS (p = 0.007) and HAM-D-17 (p = 0.011) [15]. All adverse events were mild to moderate. None of the patients in the study reported death as an outcome. Decreased appetite and dizziness were common adverse events found in the vilazodone, escitalopram, and placebo groups [16,17].

These findings support the efficacy of vilazodone (Hetero) across a broad range of depressive symptoms and severity for the treatment of MDD and indicated significant advantages over placebo in all efficacy measures. Vilazodone indicated significant antidepressant efficacy with an improved safety profile, and the difference between the vilazodone and escitalopram was not statistically significant.

## Conclusions

Vilazodone had a similar improvement in depressive symptoms on HAM-D-17, MADRS, and HAM-A scores as escitalopram. Vilazodone had statistically significant improvement in depressive symptoms on HAM-D-17, MADRS, and HAM-A scores compared to the placebo. Our study results demonstrate the efficacy, safety, and tolerability of vilazodone (10, 20, and 40mg/day), manufactured by Hetero, to treat MDD.
